# Temporary inhibition of positive phototaxis in emigratory population of  *Nilaparvata lugens* by mark-release-recapture

**DOI:** 10.1371/journal.pone.0222214

**Published:** 2019-09-06

**Authors:** Haibo Yang, Junfeng Dong, Zhenjie Hu, Dingxu Li, Fan Fang, Baoping Zhai

**Affiliations:** 1 College of Forestry, Henan University of Science and Technology, Luoyang, China; 2 College of Plant Protection, Nanjing Agricultural University, Nanjing, China; Zhejiang University, CHINA

## Abstract

Light traps are used to determine the temporal and spatial dynamics of the migratory brown planthoppers (BPHs) *Nilaparvata lugens*. But very little is known whether newly emerged adults respond to local light traps during the emigration period. Thus, it is important to evaluate the efficiency of light traps in attracting emigrant and immigrant populations to improve forecasting and control of this pest. The migration periods of *N*. *lugens* were determined by field surveys in Fuyang, Zhejiang province in 2012 and Yongfu, Guangxi Zhuang Autonomous Region in 2013. Mark-release-recapture experiments with both newly emerged (unflown) and flight experienced (flown) *N*. *lugens* were conducted at the two study sites. The marking method did not have any significant effect on the survival or flight capability of the *N*. *lugens*. A total of 4800 marked flown and 8400 unflown BPHs were released at a distance of 10, 20 and 30 m from 45-watt fluorescent actinic light traps. The results showed that without wind (< 3.2 m/s) or rainfall conditions, the overall recapture rate of flown BPHs was higher than that of unflown BPHs (9.60% and 0.92%, respectively; χ^2^_1_ = 589.66, *P* < 0.0001). Curve estimation regression analysis showed that flown BPHs were attracted to the light source at a distance of 19.77 m, and unflown BPH at a distance of 5.35 m, with these distances corresponding to a 5% recapture rate. Given that the population dynamics of BPHs in the light traps were not synchronous with that in the fields, our results indicate that only a few emerging BPHs in an infested site can be captured locally by light traps. Therefore, care must be taken in estimating the abundance of the sample to absolute local abundance during sedentary and emigration period.

## Introduction

Kennedy (1985) defined migratory behavior as a “persistent and straightened-out movement effected by the animal’s own locomotory exertions or by its active embarkation upon a vehicle. It depends on some temporary inhibition of station-keeping responses, but promotes their eventual disinhibition and recurrence.” [1]. A crucial factor in this migratory definition is the inhibition of responses to “vegetative stimuli”. During insect migration, they are not distracted from their trajectory by “vegetative stimuli” such as mates or food [2–4]. In contrast, the dispersal behavior is defined as a form of “trivial flight”, in other words, a search and foraging flight. Once the vegetative stimulus appears, the defined appetitive dispersal flight ends immediately and approaches the stimulus via an orientated search flight.

Accordingly, migratory and dispersal behaviors are distinguished by exploring the suppression of responses to resources [2]. Famous migratory animals, such as arctic terns (*Sterna paradise*) and salmon (*Oncorhynchus* and *Salmo* spp.), have been found to avoid breeding sites or food along their migration routes [5–6]. So far, the inhibition of responses to stimuli has only been studied in a few insect species. The summer migrants, *Aphis fabae* and *Brevicoryne brassicae* and the facultative migrants *Bemisia tabaci* are not affected by the presence of host-plant odor during the migratory flight in a vertical wind tunnel [7–8]. Similar results have been obtained in the bark beetles, *Scolytus multistriatus* and *Ips typographus* [9–10]. However, the temporary inhibition of station-keeping response to light during migration has not been sufficiently investigated. Therefore, we determined whether migratory brown planthoppers (BPH), *Nilaparvata lugens* temporarily inhibit their response to light traps during emigration.

Brown planthopper (BPH), *N*. *lugens* is a common rice pest found in large areas of East and Southeast Asia [11–12]. BPHs migrate annually from the Indochina Peninsula and across mainland China to the Korean Peninsula and Japan [13–16]. BPH shows wing dimorphism, a highly fecund non-flying short-winged form (brachypterous) and a migratory long-winged form (macropterous). The macropterous adults of BPH have strong positive phototaxis, thus, light traps are important to monitor seasonal fluctuations in their numbers [17]. Generally, it is easy to determine whether the light trap catches of BPH are from off-site fields during the early immigration period, but it is difficult to estimate whether light trap catches are from native habitat or distant infestation sites during the sedentary and emigration period. Previous research found that local macropterous BPHs are not trapped in light traps during the emigration period [18–19]. But there is no direct evidence for the inhibition of responses to light traps for long-range migrants such as rice planthoppers.

Only newly emerged macropterous BPHs can migrate over long distances [20]. Do BPH make a migration flight after emergence, or they simply disperse to forage for mates and food? Answering this question is of substantial relevance to the usage of light traps to control local outbreaks. Consequently, if the first flight of BPH after emergence is a true migration flight, BPH will not respond to light traps located in its native habitat. It will make a preovipository or precopulatory migration lasting for minutes or hours to new habitats. In contrast, if the first flight of BPH after emergence is a dispersal flight, BPH will immediately respond to the light traps. In this case, the efficiency of a light trap located at the site of emergence is increased.

Given the above considerations, we investigated the efficiency of light traps in rice fields to determine the proportion of marked and released BPHs was recaptured by light traps immediately after emergence. In a control experiment, the released BPHs were from previous captures by light traps, which had prior flight experiences. Therefore, two mark-release-recapture (MRR) field studies were conducted to estimate the recapture rate of flight experienced (in the following called “flown”) and newly emerged (unflown) BPHs in Zhejiang and Guangxi provinces where BPHs outbreaks are reported every year since 2012 and 2013. Our aims were to reveal the population characteristics of BPHs in light traps and facilitate the forecasting and management of these migratory pests. We determined the recapture rate of pests marked with a fluorescent dye of BPHs only under optimal conditions: in light breezy and no rainfall nights.

## Materials and methods

### Ethics statement

No specific permits were required for the described field studies. The brown planthopper *N*. *lugens* is a major pest of rice in Asia, and huge amounts of manpower and resources are used to control the damage it causes every year.

In this study, we confirmed the following: (i) the location is not privately owned or protected and (ii) the field studies did not involve endangered or protected species.

### Study area and period

This study was conducted in two locations, siteIwas set at the experimental station of China National Rice Research Institute in Fuyang (30.07°N, 119.95°E), Hangzhou, Zhejiang province; site II was at the farm of the Yongfu Plant Protection Station (24.98°N, 109.98°E), Guilin, Guangxi Zhuang Autonomous Region, China. The geographical distance between the two study sites is approximately 1200 km. The initial immigrants mainly appear in March and their progeny starts to migrate northward beginning late June in Yongfu. The immigration of *N*. *lugens* reaches the peak in mid- to late June and mid- to late July. Starting from late August, the emigrants are generally southbound in Fuyang. The farm area covered more than 20 ha of planted rice (Taichung Native 1, TN1) in both sites every year. In each location, an area of 300 × 400 m^2^ without human light sources was selected as the trapping site. Based on the actual migration period, the two MRR experiments were conducted at site I in 2012 and at site II in 2013.

### Systematic field investigation and ovary dissection

Systematic field investigations were conducted to characterize the population dynamics of BPHs in the two study sites. Each paddy in the farms of Fuyang and Yongfu was selected as the experimental. This paddy was moderately fertile and was maintained with routine cultural practices, and no pesticide was used during the rice-growing season. Systematic field investigation of BPH population was performed once every 3 d by a plant-shaking method [11, 21].

Macropterous females of BPH were collected from experimental paddies and dissected every 3 d to estimate the level of ovary development. According to the criteria used by Zheng et al. (2014) [22], the population characteristics of BPH were classified into three types as shown in Table 1.

**Table 1:** Classification of population characteristics of *Nilaparvata lugens* in paddy fields.

Type	Population characteristics	Ovarian development grades (%)
A	Immigration	Proportions of level III to level V ovarian development > 60%
B	Sedentary and local breeding	Moderate proportions of different levels of ovarian development
C	Emigration	Proportions of level I to level II ovarian development > 70%

### Adults capture and marking

Two groups of BPHs were collected in each location during sedentary and emigration periods. The first group comprised of BPHs which could not fly before being released (unflown BPHs). Fourth to fifth instar nymphs were collected by insect aspirators every 3 d. The collected nymphs were kept together in a cage (2 × 2 × 2 m) covered with a nylon screen at the experimental field. Once the adults emerged, they were immediately collected using sweep nets in the evening, i.e., at 1 h after sunset when BPH adults climb to the upper flag leaves and some fly a short distance to the wall of the cage. Insects were captured by aspirators and then marked. The second group represented BPHs which had already made a migration flight (and maybe a dispersal flight) before being released (flown BPHs). They were collected using another light trap (black light lamp, 20-watt) positioned at a long distance (4 km in Yongfu; 1 km in Fuyang) from the release site. The insects were collected in the evening during the peak periods of BPHs using light traps and insect aspirators. These BPHs might be immigrants from off-sites or long-distance migrants passing through the site. Whatever the case, they had already performed a flight before being released. Rice seedlings put in trap containers prevented excessive direct contact among the scrambling BPHs. They were transported to the release site in cooler boxes.

Micronized (3–5 μm particle size) fluorescent dye powder with three colors: scarlet, fresh green and medium yellow (Chenmei pigment masterbatch Co., Ltd., Shenzhen, China) were used to mark the insects in this study. BPH adults were aspirated into transparent plastic tubes (12 cm in length, 4 cm in diameter) containing 0.1 g of fluorescent dye powder in groups of 50. The tubes were then shaken gently by hand for 30 s to coat the BPHs with powder. This is a standard and harmless method for MRR experiments on small-sized insects and large numbers [23–24]. Different fluorescent dye powder colors were used to distinguish different release distances. The marked BPHs were immediately transferred from the plastic tubes to open petri dishes inside transparent plastic mass-rearing cages (100 cm in height, 40 cm in diameter; one for each release distance) containing TN1 rice seedlings. BPHs that flew away from the petri dishes inside the cages were counted as released. The BPHs that seemed exhausted and did not fly out were counted, and their numbers were subtracted from the total marked BPH.

### Effects of marking on BPH survival and flight activity

To ensure that the marking method used in our MRR experiments did not affect survival or flight activity of BPH adults, we examined the effect of the fluorescent dye powder on BPHs activities in laboratory conditions. A total of 280 adults of *N*. *lugens* were marked with a fluorescent dye powder with one of the three colors or were unmarked (as controls). The marked *N*. *lugens* were released into cages (100 cm in height, 40 cm in diameter) containing TN1 rice seedlings. After the release of the marked adults, the cages were placed in a rice field. The number of live treated BPHs were counted daily (the dead were removed) following the release to determine the effects of the marking method on BPHs survival.

In the *N*. *lugens* flight activity test, 144 BPHs adults were marked with red (37), green (35) and yellow (35) fluorescent dye powder, or were unmarked (37) (as controls). The treated *N*. *lugens* were fixed on their protergum with a thin pin. The adult flight behavior was observed by an infrared digital video recorder (Shanghai Mingyu Information Technology Co. Ltd. Shanghai, China). The experiment started before 6:00 pm (1 h before sunset) until the following morning. The cumulative wing-beat duration for the marked and unmarked *N*. *lugens* was recorded by video playback.

### Field experiments of the mark-release-recapture method

**Fig 1:**
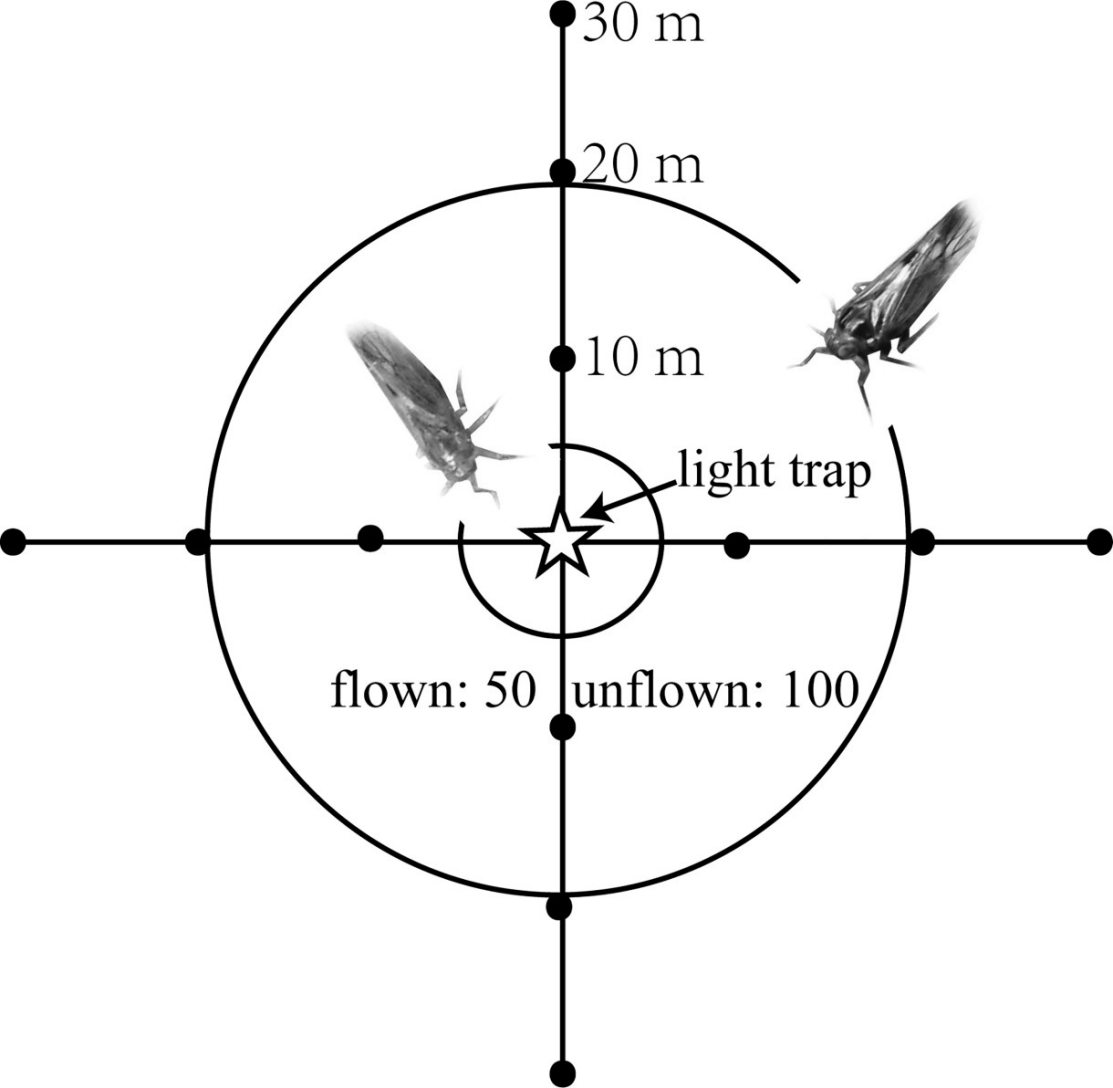
Release set-up: Dots depict the release points around light trap (star) at distances of 10, 20 and 30 m. Notes: Circles indicate attraction radii for unflown and flown BPHs, at which a minimum of 5% of individuals present are recaptured. Outer circle: Flown BPH; inner circle: Unflown BPH (see also Fig 3).

The first mark-release-recapture experiment was carried out from July 31 and continued with several breaks until September 31, in 2012 in Fuyang. Four (12 August to 10 September, flown BPHs) and three (14 to 23 September, unflown BPHs) releases of marked BPH adults were performed on different dates during the sedentary and emigration periods. The second mark-release-recapture experiment was performed from May 23 and continued with several breaks until August 5, 2013, in Yongfu. Eight releases (four: 28 May to 4 July, flown BPHs; four: 11 to 30 July, unflown BPHs) were performed on different dates during sedentary and emigration periods. The experimental layout for the release-recapture trials consisted of a light trap (fluorescent type, 45-watt actinic) positioned in the centre of rice paddy and the release areas. Marked insects were released at three distances (dyed with red at 10 m, green at 20 m and yellow at 30 m) from the light trap positioned at 2 m above ground. Fifty individuals of flown BPHs and 100 of unflown BPHs were released per release point per night. Insects were released at four directions (North, South, East, and West) around the light trap (12 release points/night) (Fig 1). The light trap was turning on automatically from 7:00 pm to 7:00 am after release in both study sites (I &II). Marked BPH was recaptured on the same day after release. All adult planthoppers collected were examined under a stereomicroscope to determine the presence of the dye every morning.

Some key meteorological parameters (temperature, relative humidity, precipitation, wind velocity, and direction) were recorded by an automatic weather station (TPJ-32-II; Zhejiang Top Instruction Co., Ltd., Hangzhou, China) during the study period. The weather station was located at approximately 50 m from the light traps. All releases took place under light breezy and dry conditions (dry, no wind, >16°C).

### Analyses

The data representing the effects of fluorescent dye powder marking on the insect survival and flight activity were subjected to analysis of variance (one-way ANOVA), and comparison of means was performed using the Tukey-Kramer honestly significant difference (HSD) test. The Pearson correlation coefficient was used to investigate the relationship between BPHs caught in light traps and fields.

We set up two types of light trap in this study, a traditional 20-watt black light trap used for collection of flown BPHs, and a 45-watt fluorescent light trap used to collect marked BPHs, and the two types of light trap were separated from each other (4 km in Yongfu, 1 km in Fuyang), but the fluorescent light trap for MRR and systematic field experiments were put in the same region. BPHs data selected from fluorescent light traps were used to compare the number of BPHs in paddies.

Given that all releases were carried out under similar favorable conditions, data from all release sessions were combined for each site. The overall effect of release direction on recaptures rates was tested by Pearson’s χ^2^-test. Since there was no difference in the relative number of recaptures originating from the four release directions, sample sizes at each of the three release distances were large enough to allow for meaningful analyses and conclusions. Curve estimations were run to model the relationship between attraction-to-light behavior and the release distance (continuous). All data were analyzed using SPSS 20 (IBM Inc., Armonk, NY, U.S.A.).

## Results

### Population characteristics of BPH in the two study sites

The population characteristics of BPH in two study sites were analyzed based on the ovarian development of female adults at different periods. The BPH populations in Fuyang fields before August 20 and in Yongfu before June 20 were type B (sedentary and local breed), with moderate ovarian development at different levels in 2012 and 2013 (Table 2). BPHs emigration occurred starting from August 21 at Fuyang and June 21 at Yongfu, and the proportion of levelIand level IIovarian development was more than 70% in both study sites.

**Table 2:** Ovarian development and population characteristics of *N*. *lugens* in paddy fields in 2012 and in 2013.

Year	Site	Date (month/day)	Ovarian development grades (%)		Population characteristics
			I-II	III-V	
2012	Fuyang	7/31-8/20	48.63	51.37	Sedentary and local breed
		8/21-9/20 9/21-9/31	76.89 76.19	23.41 23.81	Emigraton Emigraton
2013	Yongfu	5/21-6/20	43.57	56.43	Sedentary and local breed
		6/21-7/20 7/21-8/5	76.74 89.86	23.26 10.14	Emigraton Emigraton

### Effects of marking method on survival and flight activity

The mean survival days and cumulative beat duration for all treatments (including the control) are shown in Table 3. The mean survival duration for BPHs was about 12 days. There was no significant difference between the survival duration of BPHs marked with a fluorescent dye of three colors and the control (one-way ANOVA: *F* = 0.43, *df* = 3, 276, *P* > 0.05). The mean cumulative flight durations as a measure of flight activity ranged from 124 min to 138 min for all treatments and was not significantly different between the marked and unmarked BPHs (one-way ANOVA: *F* = 0.37, *df* = 3, 140, *P* > 0.05).

**Table 3:** Mean days of survival and accumulative beat duration, reflecting the effects of fluorescent dye powder marking on the survival and flight activity of *N*. *lugens*.

Color treatment	Days of survival (mean ± SE)^a^	Accumulative beat duration (min)^a^
Red	11.87 ± 0.32a	136.00 ± 8.88a
Green	12.00 ± 0.29a	124.94 ± 15.04a
Yellow	11.76 ± 0.37a	124.14 ± 14.09a
Control	12.23 ± 0.25a	138.97 ± 11.63a

^a^ Means in the same column followed by the same letters were not significantly different [Tukey-Kramer honestly significant difference (HSD) test α = 0.05]

### Comparison of population dynamics between BPH caught in light traps and fields

**Fig 2:**
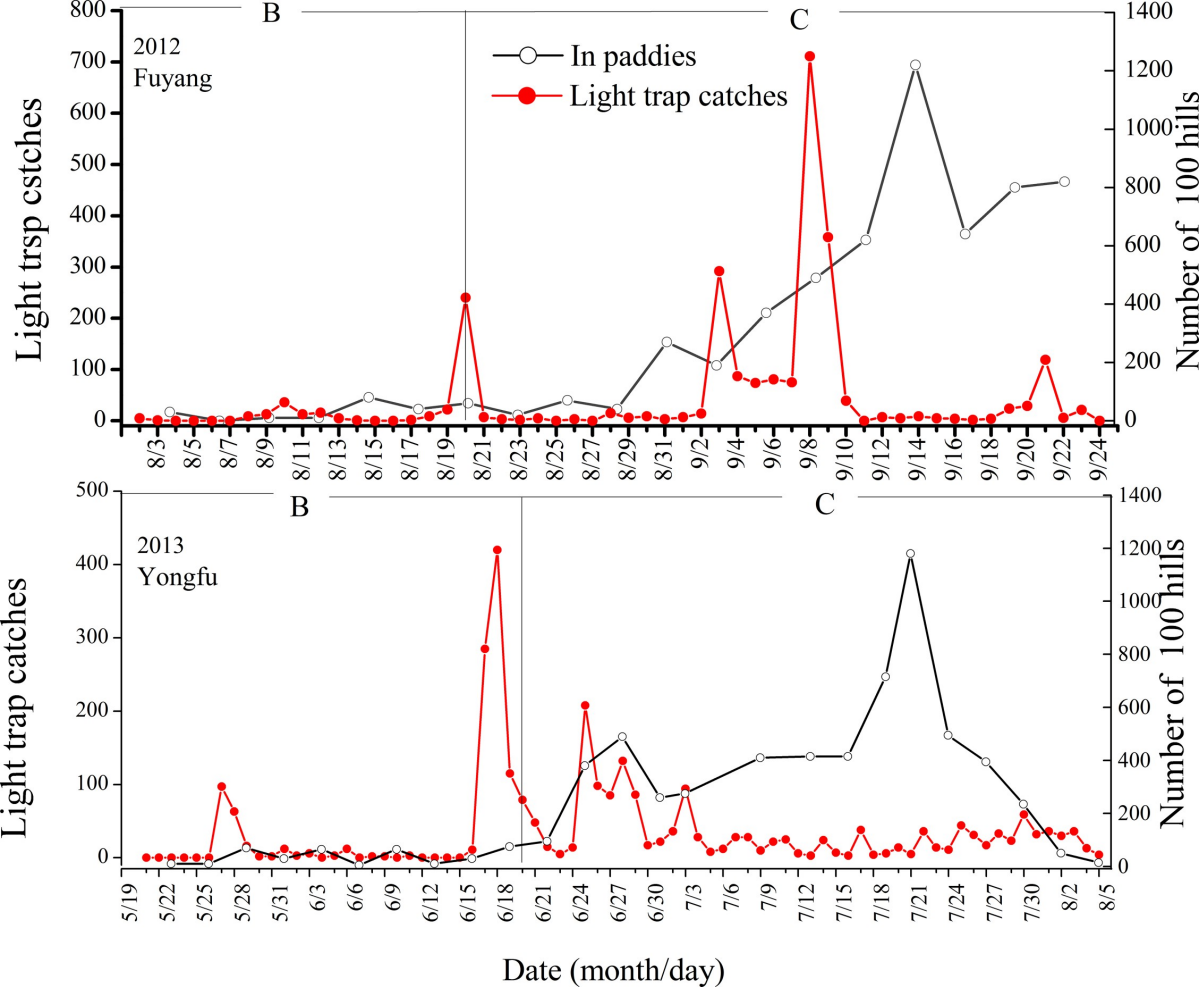
Daily fluctuation of *N*. *lugens* in light traps and in paddy fields at two study sites in 2012 and 2013. Note: B: Sedentary and local breed, C: Emigration.

The population dynamics of adult macropterous BPHs in the light traps were not consistent with those in the paddies in the two study sites (Fig 2). The light trap catches were not significantly correlated with that in paddies in Fuyang (*P* = 0.613) and Yongfu (*P* = 0.521). The main peaks of *N*. *lugens* in the light traps occurred earlier than those in the paddies in two study sites in 2012 and 2013.

In studyI, there were four main peak periods of BPH light trap catches recorded in Fuyang. The first peak period was noted in the sedentary period, while the other three peak periods occurred during the emigration period. Among these cases, the highest number of light trap catches was recorded in the third peak period (711 individuals were caught on September 8, 2012), but the population density of macropterous adults BPH in paddies was not high in this period. The population density of BPHs in the experimental paddies was low before late August and generally reached a peak in middle September. The maximum population was 1220 insects per 100 hills by September 15, 2012. However, the number of light trap catches was very small during this period.

In study siteII, there were three main peak periods of light trap catches in Yongfu. The first two peak periods occurred during the sedentary period (before June 20). The last peak period occurred during the emigration period, i.e. before the population density in the paddies reached the maximum value. The macropterous adults BPHs population density in the paddies was low before late June. The maximum population was 1180 insects per 100 hills by July 21, 2013. Similar to study siteI, the number of light trap catches was small during this period.

### Release and recapture rate

In study siteI, 2400 flown BPHs (50 insects/point × 12 points × 4 days) were released, 224 of which were recaptured. The recapture rate of marked flown BPHs ranged from 7.67 to 10.83%, and the overall recapture rate was 9.33% (95% CI, 8.20%-10.50%). Among them, 177, 40 and 7 marked BPHs were recaptured at a distance of 10, 20 and 30 m from the light trap, respectively. A total of 3600 unflown BPHs (100 insects/point × 12 points × 3 days) were released, 34 of which were recaptured. The recapture rate of marked unflown BPHs ranged from 0.92 to 1.00%, and the overall recapture rate was 0.94% (95% CI, 0.63%-1.26%). Among them, 19, 8 and 7 marked BPHs were recaptured at a distance of 10, 20 and 30 m from the light trap, respectively (Table 4).

**Table 4:** Number of recaptured marked flown and unflown BPHs at each release distance in 2012 and 2013.

Year	No. of recaptured marked flown BPH						No. of recaptured marked unflown BPH					
	Released date	10 m	20 m	30 m	Total	Recapture rate(%)	Released date	10 m	20 m	30 m	Total	Recapture rate(%)
2012	8/12	37	8	1	46	7.67						
	8/22	42	11	2	55	9.17	9/14	6	2	3	11	0.92
	9/4	55	9	1	65	10.83	9/18	8	2	2	12	1.00
	9/10	43	12	3	58	9.67	9/23	5	4	2	11	0.92
	Total	177	40	7	224	9.33	Total	19	8	7	34	0.94
	Recapture rate(%)	22.13	5.00	0.88	9.33			1.58	0.67	0.58	0.94	
2013	5/28	34	10	2	46	7.67	7/11	7	3	2	12	1.00
	6/19	51	8	1	60	10.00	7/18	5	3	2	10	0.83
	6/25	57	13	3	73	12.17	7/24	6	2	3	11	0.92
	7/4	44	12	2	58	9.67	7/30	4	4	2	10	0.83
	Total	186	43	8	237	9.88	Total	22	12	9	43	0.90
	Recapture rate(%)	23.25	5.38	1.00	9.88			1.38	0.75	0.56	0.90	

In study siteII, 2400 flown (50 insects/point × 12 points × 4 days) and 4800 unflown (100 insects/point × 12 points × 4 days) BPHs were released, 237 and 43 of which were recaptured, respectively. The recapture rate of marked flown BPHs ranged from 7.67 to 10.00% and the overall recapture rate was 9.88% (95% CI, 8.68%-11.07%). The recapture rate of marked unflown BPHs ranged between 0.83 and 1.00%, and the overall recapture rate was 0.90% (95% CI, 0.63%-1.16%) (Table 4).

Overall, the number of insects recaptured following release from the four directions was not significantly different (χ^2^_3_ = 0.69, *P* = 0.88). A total of 4800 flown (2400 + 2400) and 8400 (3600 + 4800) unflown BPHs were released at the two study sites in 2012 and 2013. The flight experienced BPHs were released for 8 days and 461 of them (9.60%) were recaptured. Of the freshly emerged BPHs, after being released for 7 days, and 77 of them (0.92%) were recaptured. The overall recapture rate of flown BPHs was about 10 times higher than the newly emerged BPHs, although a higher number of marked freshly emerged BPHs was released at the two study sites. The overall recapture rate for the flown BPHs was higher than that of unflown BPHs (χ^2^_1_ = 589.66, *P* < 0.0001) (Table 5).

**Table 5:** Recapture rate and proportion of recaptures for flown and unflown BPH at each release distance in two years.

	Flown BPH			Unflown BPH		
Distance	Releases	Recaptures	Recapture rate (%)	Releases	Recaptures	Recapture rate (%)
10m	1600	363	22.69	2800	41	1.46
20m	1600	83	5.19	2800	20	0.71
30m	1600	15	0.94	2800	16	0.57
Total	4800	461	9.60	8400	77	0.92

**Fig 3:**
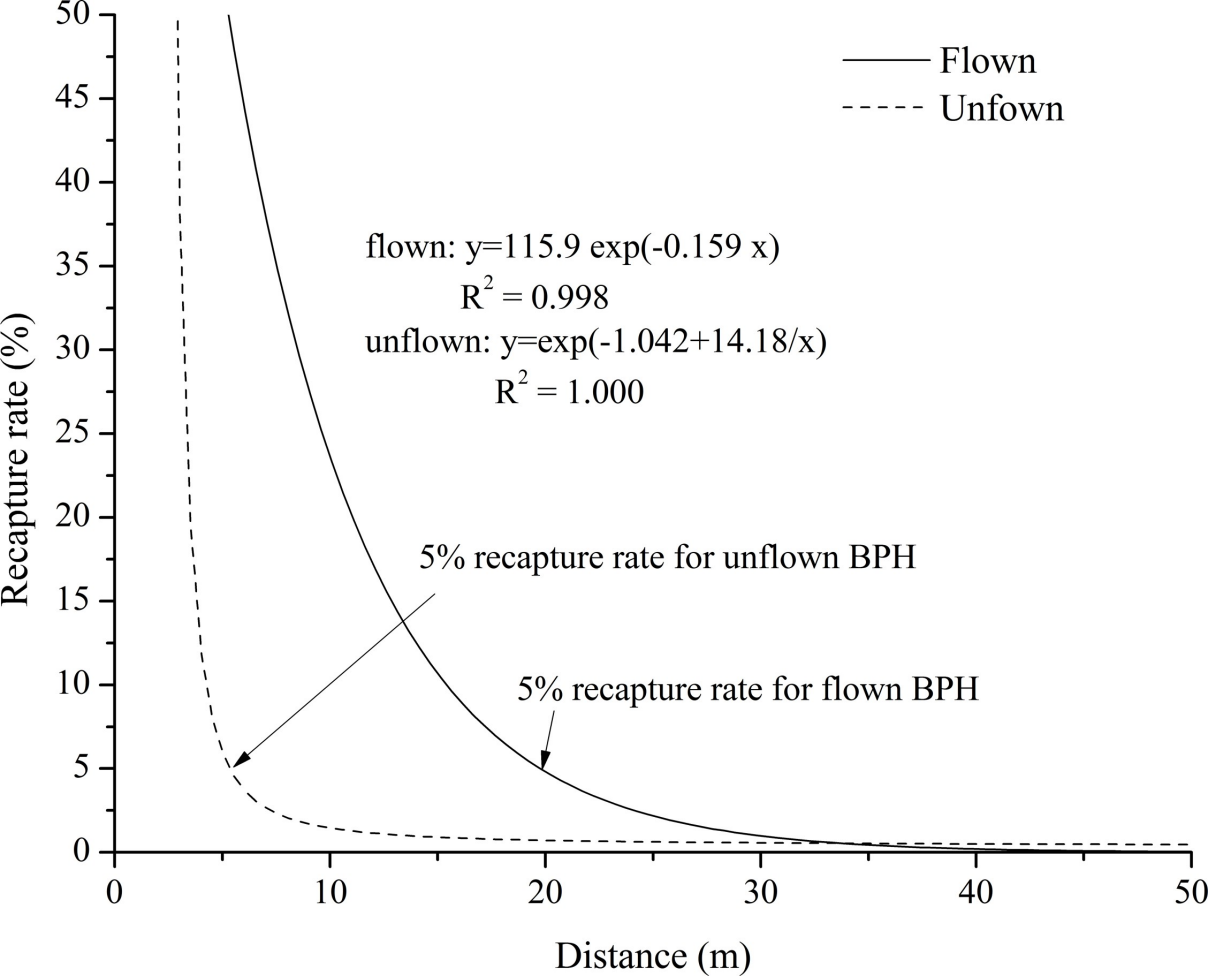
Modeled recapture rate as a function of sampling distance from the light trap. Note: The calculated distance reached by 5% of flown BPH is 19.77 m, that of unflown BPH 5.35 m.

Based on the curve estimation regression analysis, the recapture rate decreased as the distance from the light source increased (*P* < 0.05) (Table 5 and Fig 3). The regression equation for flown BPHs was y = 115.9 exp (-0.159 x), with a coefficient of determination R^2^ of 0.998 (*P* < 0.03). The regression equation for unflown BPHs was y = exp (-1.042 + 14.18/x), with a coefficient of determination R^2^ of 1.000 (*P* < 0.02) (Fig 3). The data revealed a flying-specific response in recapture rates and attraction radii: flown BPHs were attracted from up to 19.77 m, and unflown BPHs from up to 5.35 m from the light source. These specific distances corresponded to a 5% recapture rate (Figs 1 and 3).

## Discussion

This study found that there were no differences in the survival or flight activity between the undusted control BPHs and those dusted with fluorescent dye powder in laboratory conditions (Table 3). The microscopic examination of the marked insects revealed that the dye was mainly around the legs and fewer on the forewings. Among the three treatment colors of fluorescent dye, scarlet was the easiest to detect when tracking BPHs. However, the effect of this marking method on the motivation for flight is unknown.

Until the 1970s, BPHs were generally thought to be weak flies with limited power dispersal. Therefore, light traps were found to successfully attract and control local outbreaks. However, several studies have suggested that BPHs can fly over long distances covering up to hundreds of kilometers, especially with the help of wind currents [13–15]. Furthermore, light traps located at the former infested areas often attract high numbers of BPHs, indicating that light traps are highly effective [25]. However, whether such high percentages of BPHs captured were from an autochthonous population or were immigrants from distant infestation sites is not well-understood. In fact, Cheng et al. (1979) and Deng (1981) conducted surveys in an eruption area in China and found that local light traps could not trap most emigrating autochthonous adults of rice planthoppers [13, 26].

Insect migration is an adaptive mechanism that enables them to change habitats. During migration, insects are not disrupted from their routes by cues, such as food, light, color, shelter or mating partners [27]. Dispersal as ‘trivial flights’ is another mechanism that allows insects to move out of their native habitat in search of food or mates. By definition, an appetitive dispersal movement ends in the presence of stimuli, and it will be closed to the source of stimulation in an orientated search flight [10].

Therefore, a BPH with a true migration flight will not respond to light traps placed in its native habitat. The BPH will make a preovipository and precopulatory migration flight lasting for several minutes or hours, and will take the BPH to new potential breeding sites [28]. Once an adaptive migration flight ends, the BPH immediately responds to the vegetative stimuli. Cheng et al. (1979) reported that the response of autochthonous BPH to local light traps is influenced by the weather conditions of the native habitat [13]. For example, rainfall prevents BPHs to take-off from their native habitat and renders them easily attracted to local light traps. The absence of rainfall leads to the creation of trough (or “hidden peak”) of BPHs in light traps in native habitat. There must be a true peak of BPHs in light traps off-site places. Although rainfall generally inhibits insect dispersal, an increase in flight activities immediately after rainfall has been observed in horn flies [29].

Here, we report the first field experiment to assess whether autochthonous BPH adults can respond to light traps located in their native habitat. The MRR method is a powerful tool used to verify whether BPHs temporarily inhibit their station-keeping response during migration. The overall recapture rates of the marked flown and unflown BPHs in our MRR experiments in the paddy field were 9.60% and 0.92%, respectively. MRR studies on moths and midges were reported to have low recapture rates [30–31], which is in accordance with our results. These recapture rates were low compared with those reported in other insect dispersal studies that used the MRR method, e.g., 10.1% to 13.4% in a report on leafhoppers on sugarcane plants [24]. Almost all recaptured BPHs came from the central circle within 10 m away from the local light trap. In both experiments, the percentage of BPHs recaptured markedly decreased as the distance at which they were released increased. The rate of recapture was low for release distances beyond 20 m. Therefore, we inferred that the attraction radius of a light trap is short. This is expected since the visual stimulus provided by an artificial light source decreases exponentially with distance. Some marked and unmarked adults crawled up to the top of leaf blades and remained there until their take-off flight in the next morning.

Although temperature affects the number of insects caught by the light traps [32], the ambient temperature did not affect the percentages of insects recaptured in our experiments. The reason for this might be that both experiments were carried out under rather favorable environmental conditions. No experiments were carried out when the temperature was below 16°C (the minimum flight temperature of BPH). Rainfall was found to be the key factor that influenced the recapture rate of BPHs. Rainfall limits autochthonous BPHs from take-off in their native habitat and renders them more easily attracted to local light traps [13]. Fortunately, no rainfall occurred during the study period in both study sites (S1 Table).

The recapture rate of marked BPHs released in different directions may be affected by the wind direction and speed, but this possibility was not explored in this study due to low recapture rates. It should be noted that the wind direction and speed in our study are 24 hr averages and do not provide the temporal resolution to examine wind directions at night (S1 Table). Sedda et al. (2012) suggested that upwind midge flight might be a response to wind acting as a carrier of host semiochemicals, while downwind movement of midges may be due to wind transporting the midges themselves [33]. Yang et al. (2014) found that white black planthoppers *Sogatella furcifera* (Horváth) might fly against the wind at light wind nights at a speed of 0.3–1.5 m/s, whereas *N*. *lugens* might fly down at strong wind nights at a speed of 1.5–3.08 m/s [34]. There was no difference in the number of insects recaptured after being released from the four release directions under light breezy conditions, indicating that wind direction has no effect on recapture rates in the slight breeze.

The MRR experiments are aimed at explaining the fate of those insects which were not recaptured. One explanation is that they all took off from the paddy field at dusk or dawn and migrated far enough beyond the attraction radius of the light trap. The results of flight experiments in the laboratory indicated that BPHs can fly for several hours [35]. In the absence of wind, adult BPH in a straight flight will need a maximum time of 30 to 60 min to arrive at the flight boundary layer. At one incidence, a red-marked unflown BPH flew near a lamp at a distance of 200 m from its release point. A specific percentage of the released BPHs might be eaten by predators or be lost. However, for newly emerged BPHs, it is likely that they can fly beyond light effective radius before the response to light trap as opposed to stopping flying or to be killed by predators.

For flight experienced BPHs, most of them were recaptured within 10 m radius after release. The potential or even the capability to fly again was definitely reduced, because they had already flown into a light trap. The trapped BPHs consume a large amount of their energy struggling in the light trap which depletes the energy required to fly, thus we assumed that the relatively poor recapture rate of flown BPHs was due to tiredness rather than the BPHs leaving the experimental area to perform a migration flight. Furthermore, mating and sexual maturation of immigrants were suggested to occur very rapidly after landing [36]. Thus, they might be searching for a mating partner or oviposition site rather than a light trap. Besides, low percentages of recapture recorded in flown BPHs may be due to the repeated transportation process from the field to the laboratory and back. These sudden changes in environmental conditions might trigger unnatural dispersal behavior [37]. In theory, flown BPHs in addition to the stress experienced while scrambling in the traps may have prompted them to make another migration flight. Previous research has shown that some immigrants of BPHs display a re-emigration behavior [38]. This would explain the relatively high percentage of lost flown BPHs in our study.

The local macropterous adults of BPHs were not attracted by light traps. The main trap-catch peaks were not consistent with the peak periods of local macropterous adults in the field. The recapture rate was low. However, two major trap-catch peaks were observed from 8 to 9 September in 2012 in Fuyang and from 16 to 19 June in 2013 in Yongfu (Fig 2A and 2B). These peaks occurred during sedentary and emigration periods, respectively. It is hard to tell whether they were from a local population or immigrants. The first trap-catch peaks (8 to 9 September) occurred during the return migration period in Fuyang. Heavy rains occurred in the study site in these two days. This rainfall could stop BPHs from taking off and promote landing. Hence, the weather was not conducive for BPHs to take-off from this study site at the dusk of Sept. 8 and 9. Since the MRR experiments were not carried out in the rainfall conditions, we could not estimate the proportion of local BPHs that responded to the light traps. Apart from the small number of BPHs in local filed might captured by light trap as the failure of take-off, a much large of amount of BPHs must have immigrated from nearby areas if they pass the study site. According to the study site and trap-catch peak time, time–latitude profiles of wind fields along 110°E at 850 hPa at Yongfu in June were operated. During the trap-catch peaks which occurred from 16 to 19 June in Yongfu, strong Southern and Southwestern powerful winds flowing at 850 hPa were recorded (S1 Fig). Moreover, a large number of insects were captured in the light trap at or after midnight, Hence, the BPHs collected in the light traps were immigrants.

The use of light traps for mass trapping and monitoring has been successfully applied to eradicate a wide range of agricultural pests before reproduction or crop damage [32]. For any approach targeted at removing or monitoring migratory pests in fields, accurate estimation of the population characteristics of pests caught by light traps is important. The results obtained in the present study provide an approximate conclusion that local macropterous adults of BPHs may not be collected by light traps located in their native habitat if the weather conditions are appropriate for take-off, e.g. there is no rainfall and the air temperature is above 16°C in the night (S1 Table). The particular flight-to-light behavior of migratory BPH is consistent with the definition of migration.

The effectiveness of using light traps to monitor the dynamics of BPHs and control their populations remains unclear. The concept of true migration in BPH population may explain why light traps cannot completely eliminate all emerging BPHs, and why many BPHs can be captured far away from any breeding sites. The relationship between trap catches and subsequent BPHs abundance in the field remains to be established.

This study describes the first MRR experiment using flight experience and newly emerged *N*. *lugens* to determine the recapture rate and attraction radii of fluorescent pigment marked *N*. *lugens* in two most important rice-producing regions in China. Our results provide the scientific basis for the development of effective pest forecasting and control of this pest.

However, care should be taken when interpreting the results of this study. First of all, it should be noted that this study was performed under optimal conditions, and thus the relationship between the recapture rate of marked *N*. *lugens* and the abiotic factors (such as temperature, rainfall, wind, moonlight, and cloud cover) should be further analyzed in similar studies. Secondly, there is no replication in this study. It was difficult to obtain *N*. *lugens*, especially flown BPHs, because of the number of released BPH adults in the light traps gradually decreased. Thirdly, the weight of fluorescent pigment on the body of BPH individuals was not explored in this study. Notwithstanding these limitations, this study suggests that the response of emigrants of BPHs to local light traps is inhibited.

## Supporting information

S1 Fig Time–latitude profiles of wind fields along 110°E at 850 hPa at Yongfu in June 2013. (Dotted box means trap-catch peak areas of BPH).
(TIF)

S1 Table The meteorological parameters during the MRR period in both study sites. (DOCX)
