# Supplementary figures and images for: Temporary inhibition of positive phototaxis in emigratory population of Nilaparvata lugens by mark-release-recapture
Source: PLoS One. 2019 Sep 6;14(9):e0222214. doi: 10.1371/journal.pone.0222214 (PMC6730993; doi:10.1371/journal.pone.0222214)

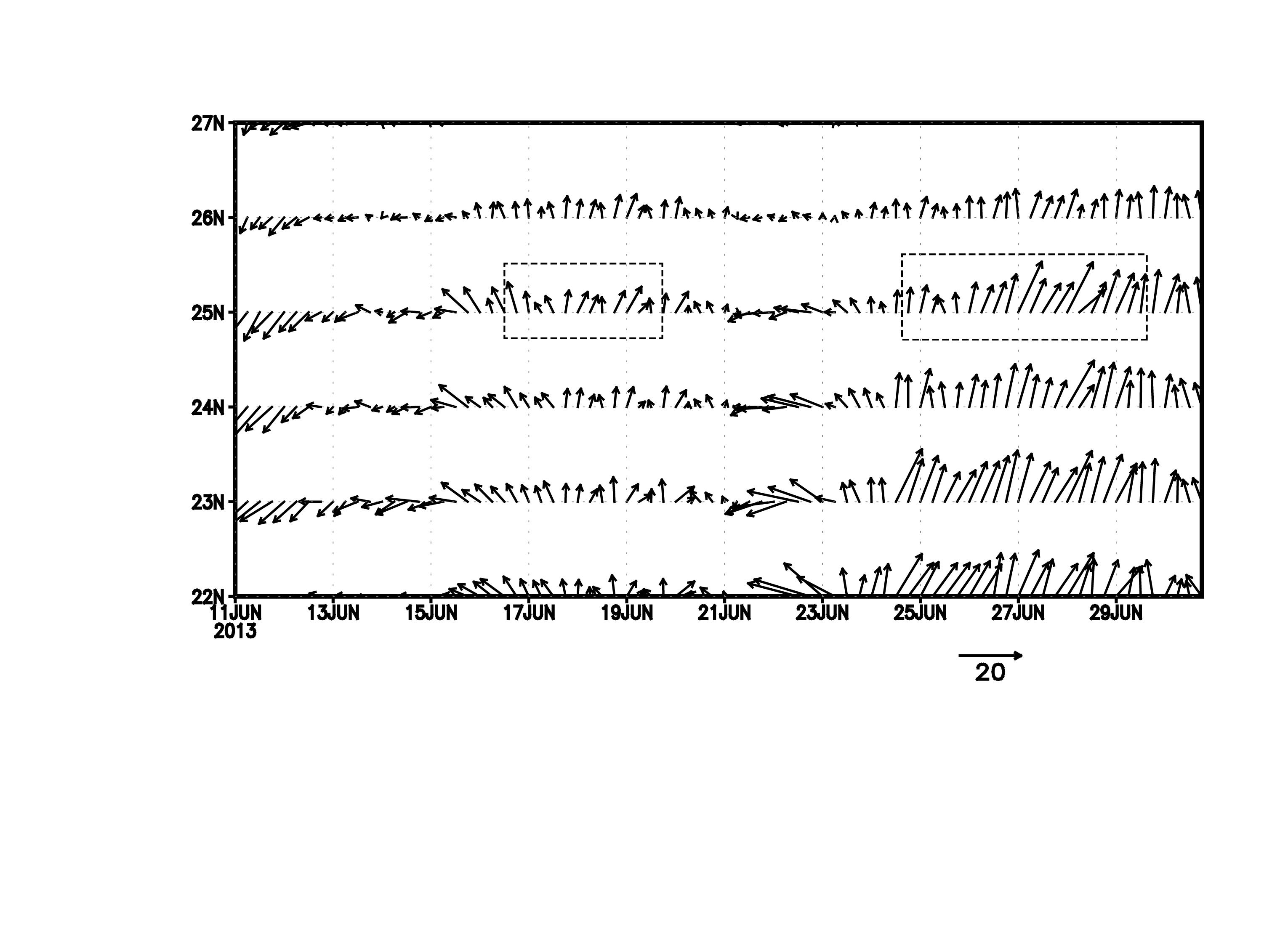

Supplement: S1 Fig — (Dotted box means trap-catch peak areas of BPH). (TIF) [file pone.0222214.s001.tif]
