# Supplementary material for: Temporary inhibition of positive phototaxis in emigratory population of Nilaparvata lugens by mark-release-recapture
Source: PLoS One. 2019 Sep 6;14(9):e0222214. doi: 10.1371/journal.pone.0222214 (PMC6730993; doi:10.1371/journal.pone.0222214)
Supplement: S1 Table — (DOCX) [file pone.0222214.s002.docx]

**S1 Table. The meteorological parameters during the MRR period in both study sites.**

| Year | Recapture date | Temperature (℃) | Relative humidity | Precipitation (mm) | Wind velocity (m/s) | Wind direction |
| --- | --- | --- | --- | --- | --- | --- |
| 2012 | 8/12 | 24.24-34.05 | 60.80-98.70 | 0 | 0.0-0.5 | W |
|  | 8/22 | 24.18-33.71 | 85.50-90.10 | 0 | 0.1-1.0 | NE |
|  | 9/4 | 18.89-24.43 | 71.20-90.10 | 0 | 0.0-2.2 | N |
|  | 9/10 | 21.64-26.03 | 77.58-99.20 | 0 | 0.1-2.0 | NE |
|  | 9/14 | 17.56-23.72 | 68.94-99.50 | 0 | 0.2-2.0 | NE |
|  | 9/18 | 16.95-25.49 | 60.25-98.30 | 0 | 0.0-2.0 | NW |
|  | 9/23 | 18.23-25.10 | 72.61-96.70 | 0 | 0.0-2.0 | No regular |
| 2013 | 5/28 | 24.31-33.25 | 78.62-84.73 | 0 | 0.3-2.2 | S |
|  | 6/19 | 25.50-34.42 | 66.24-86.34 | 0 | 0.5-2.0 | S |
|  | 6/25 | 24.81-33.03 | 82.64-96.93 | 0 | 0.1-3.2 | S |
|  | 7/4 | 25.31-33.24 | 60.72-85.24 | 0 | 0.9-3.2 | S |
|  | 7/11 | 24.24-34.41 | 53.47-93.57 | 0 | 0.0-2.0 | S |
|  | 7/18 | 25.04-34.12 | 57.34-83.57 | 0 | 0.0-1.0 | SW |
|  | 7/24 | 24.71-34.83 | 56.86-86.94 | 0 | 0.2-3.2 | S |
|  | 7/30 | 24.64-35.23 | 50.35-80.37 | 0 | 0.0-1.0 | S |
